# Multiplex-PCR Identification of Positive Blood Cultures: Pathogen Epidemiology, Resistance Determinants, and the Economic and Interpretive Cost of Qualitative Rapid Diagnosis

**DOI:** 10.3390/diagnostics16142252

**Published:** 2026-07-18

**Authors:** Laura Lupo, Ilaria Serafini, Daniela Chirizzi, Angela Spedicato, Gabriele Bianco, Daniele Pisanò, Maria Rita Orsi, Giovanni Moschettini, Marcella Marullo, Angelo Sorge, Rosanna Bruno, Camilla Panico, Silvana Arnesano, Antonella Simone, Luciana Corciulo, Antonella Pico, Claudia Pagano, Letizia Fulceri, Giulia Apruzzi, Anna De Filippis, Massimiliano Galdiero, Francesco Broccolo

**Affiliations:** 1 UOSD, Microbiologia e Virologia Universitaria, P.O. “Vito Fazzi”, ASL Lecce, 73100 Lecce, Italyantonella.pico@asl.lecce.it (A.P.);; 2 Department of Experimental Medicine (DiMeS), University of Salento, 73100 Lecce, Italy; 3 UOC Hospital Pharmacy, P.O. Vito Fazzi, ASL Lecce, 73100 Lecce, Italy; 4 Dipartimento di Medicina Sperimentale, Università degli Studi della Campania “Luigi Vanvitelli”, 80138 Napoli, Italy; 5 UOC Microbiologia e Virologia, AOU “Luigi Vanvitelli”, 80138 Napoli, Italy

**Keywords:** bloodstream infection, sepsis, FilmArray BCID2, syndromic multiplex PCR, antimicrobial resistance, diagnostic stewardship, cost analysis, rapid diagnostics, polymicrobial bacteraemia

## Abstract

***Background/Objectives:*** Bloodstream infections (BSIs) carry high mortality, and any delay in initiating optimal antimicrobial therapy worsens outcomes. Syndromic multiplex-PCR panels applied to signal-positive blood cultures markedly shorten time-to-identification but are expensive and report results in a strictly qualitative format. We describe twenty-two months of real-world BioFire FilmArray Blood Culture Identification 2 (BCID2) testing in a southern Italian hospital, focusing on pathogen epidemiology, resistance-gene detection, and the economic and interpretive trade-offs of the assay. ***Methods:*** We retrospectively reviewed all BCID2 runs performed on signal-positive blood-culture bottles at P.O. “Vito Fazzi” (ASL Lecce) from July 2024 to April 2026. Each run was classified as single-pathogen-positive, polymicrobial (co-infection)-positive, or negative (no on-panel target). Resistance determinants were tabulated by year and by presumptive carrier. A direct-cost analysis was performed at a reagent cost of €250 per test. ***Results:*** Of 498 runs, 401 (80.5%) yielded at least one on-panel target (330 single-pathogen, 71 polymicrobial), whereas 97 (19.5%) were negative. Among 526 organism detections, Gram-positive (48.9%) and Gram-negative (45.8%) bacteria predominated; *Staphylococcus* spp., *Escherichia coli* and the *Klebsiella pneumoniae group* were most frequent. Resistance determinants (*n* = 244) were dominated by *mecA/C*, *CTX-M* and *KPC*. Co-infections (17.7% of positives) frequently prevented unambiguous attribution of a resistance gene to a specific co-detected organism. Total reagent expenditure was €124,500, of which €24,250 (19.5%) was spent on negative results. ***Conclusions:*** BCID2 delivered rapid, broad pathogen and resistance-gene detection in a high-resistance setting. Its qualitative-only output, the difficulty of attributing a resistance gene to a specific organism in polymicrobial samples, and the cost of negative results nonetheless argue for diagnostic-stewardship gating, semiquantitative reporting, and local validation of complementary and lower-cost assays.

## 1. Introduction

Bloodstream infections (BSIs) and sepsis remain among the most time-critical emergencies in hospital medicine, and each hour of delay in administering an active antimicrobial agent is associated with measurable increases in mortality [1]. The microbiological diagnosis of BSI has historically relied on a sequential workflow: a blood-culture bottle flags positive in an automated incubator, a Gram stain is performed, and the organism is then identified and characterised for antimicrobial susceptibility over the following 24–72 h. During this interval clinicians are obliged to maintain broad-spectrum empirical therapy, with the attendant risks of toxicity, *Clostridioides difficile* infection, and selection of resistant flora.

Syndromic multiplex polymerase chain reaction (PCR) panels applied directly to signal-positive blood cultures were introduced to compress this interval. The BioFire FilmArray Blood Culture Identification 2 (BCID2) panel (bioMérieux, Salt Lake City, UT, USA) is a fully automated, nested multiplex-PCR assay run directly on a positive bottle. It interrogates 43 molecular targets, namely 11 Gram-positive bacteria, 15 Gram-negative bacteria, seven yeasts and 10 antimicrobial-resistance (AMR) genes, and returns a result in approximately one hour [2,3,4]. Multi-centre and regulatory studies report overall sensitivity and specificity exceeding 98% for on-panel targets [2,5], with comparable accuracy for the detection of *Candida* species [6], and several pre/post studies have shown that, when results are coupled with an antimicrobial-stewardship programme (ASP), BCID2 shortens the time to optimal therapy and may improve outcomes [7,8,9,10,11].

These advantages come with several trade-offs. The reagent cost of the assay is high, of the order of €250 per test in our procurement framework, so the panel cannot be applied indiscriminately without a defensible economic rationale. Because the assay is run on bottles that have already signalled positive, a negative BCID2 result is not equivalent to a negative blood culture: it indicates that the organism that grew is not represented on the panel (an off-panel target), is present below the limit of detection, or that the bottle signal was artefactual. Such results still consume a full reagent. For the clinician at the bedside the central limitation is that the panel reports targets in a strictly qualitative “detected/not detected” format. It provides neither an organism load nor a cycle-threshold value, and in polymicrobial specimens it cannot indicate which co-detected organism is the dominant pathogen or which organism carries a detected resistance gene [2,4,8].

Southern Italy is a high-prevalence setting for carbapenem-resistant and extended-spectrum β-lactamase (ESBL)-producing *Enterobacterales*, which raises the clinical stakes of rapid genotypic resistance detection but also amplifies the interpretive ambiguity described above. We here report twenty-two months of routine BCID2 use at P.O. “Vito Fazzi” (ASL Lecce), with three aims: (i) to describe the pathogen and resistance-gene epidemiology revealed by the panel; (ii) to quantify the direct economic burden of the strategy, including the cost of negative results; and (iii) to appraise the interpretive limitations of a qualitative assay and the consequent need for semiquantitative reporting and for local validation of complementary tests. Importantly, this is a clinical-utility and health-economic appraisal of routine practice, not a re-validation of the panel’s diagnostic accuracy, which is already established in the literature; in keeping with national and international consensus protocols the panel was applied only to signal-positive blood-culture bottles, as a rapid adjunct to conventional culture rather than as a culture-independent or stand-alone diagnostic test.

## 2. Materials and Methods

### 2.1. Setting and Study Period

**Figure 1:**
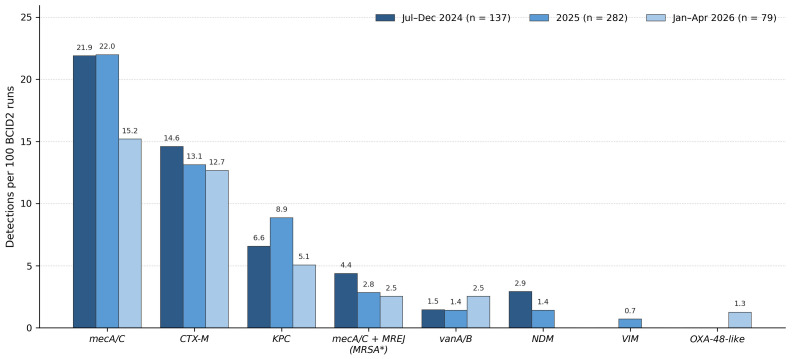
Antimicrobial-resistance determinants detected by the BCID2 panel, expressed as detections per 100 runs to control for the unequal observation windows (137, 282 and 79 runs in the three segments, respectively). MRSA* denotes the *mecA/C*-with-*MREJ* signature reported only in *S. aureus*-containing specimens. The genes *IMP* and *mcr-1* were never detected in any calendar segment.

This retrospective, single-centre study was conducted at the UOSD Microbiologia e Virologia Universitaria of P.O. “Vito Fazzi”, ASL Lecce (Apulia, southern Italy), a tertiary referral hospital serving the province of Lecce. All BCID2 assays performed on signal-positive blood-culture bottles between 1 July 2024 and 30 April 2026 were included, spanning three calendar segments, July–December 2024 (6 months), the whole of 2025 (12 months) and January–April 2026 (4 months), for a cumulative observation window of twenty-two months. All consecutive signal-positive bottles flagged during the study period were processed with BCID2. Only adult patients were included, and paediatric cases were excluded. For the analysis, only the first bloodstream-infection episode per patient was retained: repeat positive bottles from the same patient and duplicate isolates were excluded, so the epidemiological counts are not biased by repeated sampling from individual patients. The three calendar segments are of unequal length because they reflect the date of routine introduction of the panel (July 2024) and the data-extraction cut-off (April 2026) rather than a pre-specified sampling scheme; to control for the unequal denominators, temporal comparisons are expressed per 100 runs (Figure 1).

### 2.2. Blood Culture and BCID2 Workflow

Blood-culture bottles were incubated in an automated continuous-monitoring blood-culture system (BACT/ALERT VIRTUO, bioMérieux, Marcy-l’Étoile, France). Upon a positive signal, an aliquot was processed with the BioFire FilmArray BCID2 panel on a FilmArray Torch module (bioMérieux, Salt Lake City, UT, USA) according to the manufacturer’s instructions, in parallel with conventional Gram stain, subculture and downstream identification (bioMérieux VITEK MS, Marcy-l’Étoile, France) and phenotypic antimicrobial-susceptibility testing (VITEK 2, bioMérieux, Marcy-l’Étoile, France) as standard of care. The 43 BCID2 targets comprise Gram-positive bacteria (*Enterococcus faecalis*, *E. faecium*, *Listeria monocytogenes*, *Staphylococcus* spp., *S. aureus*, *S. epidermidis*, *S. lugdunensis*, *Streptococcus* spp., *S. agalactiae*, *S. pneumoniae*, *S. pyogenes*), Gram-negative bacteria (*Acinetobacter calcoaceticus–baumannii complex*, *Bacteroides fragilis*, *Enterobacterales*, *Enterobacter cloacae complex*, *Escherichia coli*, *Klebsiella aerogenes*, *K. oxytoca*, *K. pneumoniae* group, *Proteus* spp., *Salmonella* spp., *Serratia marcescens*, *Haemophilus influenzae*, *Neisseria meningitidis*, *Pseudomonas aeruginosa*, *Stenotrophomonas maltophilia*), seven yeasts (*Candida albicans*, *C. auris*, *C. glabrata*, *C. krusei*, *C. parapsilosis*, *C. tropicalis*, *Cryptococcus neoformans/gattii*) and ten AMR genes (*mecA/C*, *mecA/C* and *MREJ* [MRSA], *vanA/B*, *CTX-M*, *IMP*, *KPC*, *NDM*, *OXA-48-like*, *VIM*, *mcr-1*). The turnaround time from bottle positivity to an available result was approximately one hour for the BCID2 panel, compared with local turnaround for conventional methods, 24–48 h to identification and 48–72 h to full susceptibility for conventional identification and susceptibility testing.

### 2.3. Definitions and Data Extraction

For each run, results were classified as: (a) single-pathogen-positive, i.e., one on-panel organism detected; (b) polymicrobial (co-infection)-positive, i.e., two or more on-panel organisms detected in the same specimen; or (c) negative, i.e., no on-panel target detected. Genus-level targets (*Staphylococcus* spp., *Streptococcus* spp.) and the family-level *Enterobacterales* target were recorded as reported by the instrument. Detections of each organism and each resistance gene were aggregated by calendar segment. For every resistance gene, the presumptive bacterial carrier was inferred from the co-detected organism(s) on the same report, with the explicit caveat that the assay does not establish this linkage genetically in polymicrobial specimens (Section 4.3). Reconciliation of on-panel results against conventional culture was not undertaken, because diagnostic-accuracy validation was not an objective of this study: the analytical and clinical performance of the BCID2 panel on signal-positive bottles is already established, and in our laboratory the panel is used only as a rapid identification adjunct to a conventional culture pathway (Gram stain, subculture and phenotypic susceptibility testing) that runs in parallel on every bottle and remains the basis for definitive therapy. Off-panel organisms are consequently handled by that parallel pathway rather than by the panel. To avoid inflating organism frequencies through polymicrobial specimens, pathogen occurrence is reported against the number of specimens (*n* = 401 positive runs) alongside total detections; a patient-level denominator was not available from the anonymised aggregate records.

### 2.4. Direct-Cost Analysis

A direct-cost analysis was performed from the laboratory’s perspective using the per-test reagent acquisition cost of €250. Total expenditure was computed as the number of runs multiplied by the unit cost, and was disaggregated by calendar segment and by outcome (positive vs. negative). The expenditure attributable to negative runs was reported separately as the principal indicator of the “cost of negative results.” This is therefore a direct reagent-cost appraisal and not a full health-economic evaluation: capital equipment depreciation, instrument maintenance and service contracts, staff time, and downstream effects on length of stay and antibiotic consumption were not costed. These components are considered qualitatively with reference to the published literature in Section 4.1, and are listed among the study’s limitations (Section 4.6).

### 2.5. Statistical Analysis and Ethics

Categorical variables are summarised as counts and proportions with 95% confidence intervals calculated by the Wilson method. Proportions were compared across the three calendar segments using the *χ*^2^ test (Fisher’s exact test where expected cell counts were small) and the Cochran–Armitage test for linear trend; a two-sided *p* < 0.05 was considered statistically significant. Descriptions of temporal “stability” are used only for comparisons that were not statistically significant on these tests. Per-gene resistance counts by calendar segment were too small to support formal trend testing and are presented descriptively, normalised per 100 runs (Figure 1). Analyses were performed in R v4.3.1 (R Foundation for Statistical Computing, Vienna, Austria).

The study was conducted in accordance with the Declaration of Helsinki. It was a strictly retrospective analysis of fully anonymised, aggregated laboratory data generated during routine diagnostic activity over the study period: no change was made to any patient’s clinical management, no additional or prospective testing was performed, and no individually identifiable data were processed, the work consisting solely of the statistical processing of results already produced by routine analyses. In our setting, retrospective analyses of anonymised, aggregated routine diagnostic data of this kind do not require formal ethics-committee approval.

During the preparation of this manuscript the authors used a large-language-model assistant for the purposes of drafting, tabulation of de-identified aggregate counts, and language editing. The authors have reviewed and edited the output and take full responsibility for the content of this publication.

## 3. Results

### 3.1. Testing Volume, Positivity and Co-Infection

A total of 498 BCID2 runs were performed over the study period: 137 in the second half of 2024, 282 in 2025 and 79 in the first four months of 2026. An on-panel target was detected in 401 runs (80.5%; 95% CI 76.8–83.8), of which 330 (66.3% of all runs) were single-pathogen and 71 (14.3% of all runs) were polymicrobial. The remaining 97 runs (19.5%; 95% CI 16.2–23.2) returned no on-panel target. Overall positivity did not differ significantly across the three calendar segments (78.1%, 81.6% and 81.0%; χ^2^ = 0.72, df = 2, *p* = 0.70; Cochran–Armitage test for trend *p* = 0.52), and the proportion of polymicrobial results among positive runs was likewise stable (17.8%, 17.8% and 17.2%; 17.7% overall, 95% CI 14.3–21.7; χ^2^ = 0.01, df = 2, *p* = 0.99; trend *p* = 0.94). The volume and outcome distribution are summarised in Table 1.

### 3.2. Pathogen Epidemiology

The 401 positive runs yielded 526 organism detections, equivalent to 1.31 targets per positive specimen. Gram-positive bacteria accounted for 257 detections (48.9%), Gram-negative bacteria for 241 (45.8%) and yeasts for 28 (5.3%). Among Gram-positive targets, the genus-level *Staphylococcus* spp. result dominated (133 detections), followed by *S. epidermidis* (50) and *S. aureus* (20); enterococci and streptococci were less frequent. Among Gram-negative targets, *Escherichia coli* was the single most common organism overall (95 detections), followed by the *Klebsiella pneumoniae group* (67) and *Pseudomonas aeruginosa* (31). *Candida* species accounted for all yeast detections, led by *C. albicans* (11). Ten panel targets (including *Listeria monocytogenes*, *S. lugdunensis*, *S. pyogenes*, *Salmonella* spp., *Haemophilus influenzae*, *Neisseria meningitidis*, *Candida auris*, *C. krusei* and *Cryptococcus neoformans/gattii*) were never detected. Detections by organism and segment are reported in Table 2.

**Table 2:** On-panel organism detections by group and calendar segment (only targets detected at least once are listed).

Target	2024	2025	2026	Total	% org.
***Gram-positive bacteria***					
*Staphylococcus* spp.	38	83	12	133	25.3
*Staphylococcus epidermidis*	14	31	5	50	9.5
*Staphylococcus aureus*	7	11	2	20	3.8
*Enterococcus faecalis*	4	10	2	16	3.0
*Streptococcus* spp.	4	9	3	16	3.0
*Enterococcus faecium*	3	7	2	12	2.3
*Streptococcus pneumoniae*	2	4	3	9	1.7
*Streptococcus agalactiae (GBS)*	0	1	0	1	0.2
*Subtotal Gram-positive*	72	156	29	257	48.9
***Gram-negative bacteria***					
*Escherichia coli*	28	54	13	95	18.1
*Klebsiella pneumoniae group*	18	39	10	67	12.7
*Pseudomonas aeruginosa*	9	18	4	31	5.9
*Enterobacter cloacae complex*	3	6	2	11	2.1
*Proteus* spp.	4	5	2	11	2.1
*Stenotrophomonas maltophilia*	3	5	3	11	2.1
*Klebsiella oxytoca*	2	4	2	8	1.5
*Serratia marcescens*	2	2	0	4	0.8
*Acinetobacter calcoaceticus–baumannii complex*	1	1	0	2	0.4
*Bacteroides fragilis*	1	0	0	1	0.2
*Subtotal Gram-negative*	71	134	36	241	45.8
***Yeasts***					
*Candida albicans*	3	6	2	11	2.1
*Candida glabrata*	2	3	2	7	1.3
*Candida parapsilosis*	1	4	2	7	1.3
*Candida tropicalis*	1	1	1	3	0.6
*Subtotal yeasts*	7	14	7	28	5.3
*Total organism detections*	150	304	72	526	100

GBS, group B *Streptococcus*; % org., percentage of all 526 organism detections. The family-level *Enterobacterales* target co-reported with the listed *Enterobacterales* species and is not counted separately. Subtotal and total rows are aggregates. This table provides the complete per-organism count; the detected resistance determinants and their presumptive carriers are summarised in Table 3.

**Table 3:** Antimicrobial-resistance determinants detected by the BCID2 panel, by calendar segment and presumptive carrier, with the associated resistance phenotype and inferred therapeutic implication.

Determinant	2024	2025	2026	Total	Presumptive Carrier	Resistance Phenotype and Inferred Therapeutic Implication
*mecA/C*	30	62	12	104	*S. epidermidis* and *Staphylococcus* spp.	Methicillin/β-lactam resistance in staphylococci; anti-staphylococcal β-lactams ineffective—use a glycopeptide, daptomycin or ceftaroline
*CTX-M*	20	37	10	67	*E. coli* and *K. pneumoniae* group	ESBL (third-generation cephalosporin resistance); carbapenem, or a carbapenem-sparing regimen under stewardship
*KPC*	9	25	4	38	*K. pneumoniae* group	Class A carbapenemase; ceftazidime–avibactam, meropenem–vaborbactam or imipenem–relebactam
*mecA/C* + *MREJ* (MRSA)	6	8	2	16	*S. aureus* only	Methicillin resistance confirmed in *S. aureus*; anti-MRSA agent (glycopeptide, daptomycin, ceftaroline), β-lactams excluded
*vanA/B*	2	4	2	8	*E. faecium* only	Glycopeptide resistance in enterococci; linezolid or daptomycin
*NDM*	4	4	0	8	*E. coli*; *K. pneumoniae* group	Metallo-β-lactamase; ceftazidime–avibactam plus aztreonam, or cefiderocol
*VIM*	0	2	0	2	*P. aeruginosa*	Metallo-β-lactamase; cefiderocol, or an aztreonam-based combination
*OXA-48-like*	0	0	1	1	*K. pneumoniae* group (with *CTX-M*)	Class D carbapenemase; ceftazidime–avibactam
**Total**	**71**	**142**	**31**	**244**	—	—

Determinants are listed in descending order of total detections; counts are reproduced across calendar segments. *IMP* and the colistin-resistance gene *mcr-1* were sought but never detected. Therapeutic implications are general inferences from the detected genotype and from regional susceptibility patterns and are not a substitute for phenotypic antimicrobial-susceptibility testing, which remained the basis for definitive therapy and de-escalation. In polymicrobial specimens a determinant cannot be attributed to a specific co-detected organism (Section 4.3).

### 3.3. Polymicrobial Specimens and the Attribution Problem

Polymicrobial detections (71 specimens) were not randomly distributed across taxa but clustered in two recurrent patterns. The first was a staphylococcal pattern, in which the genus-level *Staphylococcus* spp. target co-reported systematically with *S. epidermidis* and/or *S. aureus*; in these specimens *mecA/C* was almost invariably present, and MRSA-defining *mecA/C* with *MREJ* was reported when *S. aureus* was a constituent. The second was an *Enterobacterales* pattern, in which the family target co-reported with *E. coli*, *K. pneumoniae* group, *Proteus* spp., *Enterobacter cloacae complex* or *K. oxytoca*, frequently together with one or more β-lactamase genes. A minority of specimens contained complex mixed clusters spanning enterococci, staphylococci and Gram-negative organisms. In all such specimens the instrument reported the resistance gene(s) without specifying the carrier, so that the gene-to-organism linkage had to be inferred (Section 4.3).

### 3.4. Antimicrobial-Resistance Determinants

A total of 244 resistance-gene detections were recorded (71 in 2024, 142 in 2025, 31 in 2026). The methicillin-resistance determinant *mecA/C* was the most frequent (104 detections), reflecting the high burden of staphylococci and, in particular, of methicillin-resistant *S. epidermidis*; the MRSA-specific *mecA/C*-with-*MREJ* signature accounted for a further 16 detections, all in specimens containing *S. aureus*. The ESBL determinant *CTX-M* (67 detections) and the carbapenemase *KPC* (38 detections) were the dominant Gram-negative resistance markers, principally in *E. coli* and the *K. pneumoniae* group, broadly in keeping with, although not formally compared against, regional surveillance for southern Italy. Vancomycin resistance (*vanA/B*, eight detections) was confined to *E. faecium*; the metallo-β-lactamases *NDM* (8) and *VIM* (2) and a single *OXA-48-like* detection completed the carbapenemase picture, while *IMP* and the colistin-resistance gene *mcr-1* were never detected (Table 3). Normalised for the unequal observation windows (detections per 100 BCID2 runs), the rank order of the dominant determinants was preserved across the three segments (no formal trend test was applied, given the small per-gene counts), with *mecA/C* and *CTX-M* consistently dominant and the carbapenemases concentrated in 2024–2025 (Figure 1).

### 3.5. Economic Analysis

At a unit reagent cost of €250, the 498 runs represented a total direct expenditure of €124,500 over twenty-two months. Of this, €100,250 (80.5%) was spent on the 401 runs that returned an actionable pathogen or resistance target, while €24,250 (19.5%) was spent on the 97 negative runs. Expenditure scaled with volume across the three segments (€34,250 in 2024, €70,500 in 2025 and €19,750 in 2026). The cost of negative results was therefore stable and non-trivial, corresponding to roughly one in five reagent euros (Table 4). Expressed per informative result, the cost of one actionable (positive) report was €310 (€124,500/401 runs), the gap from the €250 unit price reflecting the reagent spend absorbed by negative runs.

## 4. Discussion

Over twenty-two months of routine use, the BCID2 panel returned an on-panel pathogen in four of every five signal-positive bottles and detected a clinically relevant resistance gene in a substantial minority of specimens, within roughly one hour of bottle positivity. The epidemiological picture, namely staphylococcus-dominated Gram-positive disease, an *E. coli*/*K. pneumoniae*-led Gram-negative profile, and a *CTX-M*/*KPC*-dominated resistance landscape, is internally coherent and consistent with the high endemic burden of ESBL- and carbapenemase-producing *Enterobacterales* described for southern Italy. The clinical value of obtaining this information one to three days earlier than conventional methods is well documented [7,8,9,10,11]. Three features of our experience deserve closer scrutiny: the economic structure of the strategy, the interpretive limits of a qualitative assay, and the consequent need to validate complementary tests.

### 4.1. Health-Economic Considerations and the Cost of Negative Results

The economic case for syndromic blood-culture panels does not rest on reagent cost, which at €250 per run is an order of magnitude higher than the consumables of conventional identification, but on downstream savings. Published pre/post and randomised data, especially when results are coupled with an ASP, show reductions in time to optimal therapy of the order of 17 to 34 h, shorter time to de-escalation, and, in some series, reduced 30-day mortality and length of stay [7,9,10,11]; the rapid recognition of contaminant coagulase-negative staphylococci alone has been associated with appreciable savings in post-culture length of stay [12]. Against these potential benefits, the present analysis quantifies the component that is most often overlooked: the €24,250 (19.5% of total spend) consumed by runs that returned no on-panel target. A negative BCID2 result on a positive bottle still carries information, since it argues against the panel’s targets and may redirect the work-up toward off-panel organisms, but it does not by itself direct pathogen-specific therapy and it costs the same as a fully informative run. This is what we mean by the “cost of negative results”, and it is the main argument for diagnostic-stewardship gating: restricting reflexive panel use to clinical scenarios in which a rapid genotypic answer will change management (e.g., suspected Gram-negative sepsis in a high-resistance unit, or neutropenic fever), rather than testing every positive bottle indiscriminately.

These reagent costs are best weighed against the downstream costs they can avert rather than read in isolation. In our system a single avoidable inpatient day on a general ward is of the order of €600–800, and an intensive-care day several times that; the incremental cost of an inappropriate or unnecessarily prolonged broad-spectrum regimen, together with the management of an avoidable episode of *Clostridioides difficile* infection or of a resistant superinfection, is of comparable magnitude. On these figures, a strategy that—coupled with stewardship—shortens time to optimal therapy by 17–34 h and avoids even a fraction of an inpatient day in a minority of the 401 actionable cases [7,9,10,11] would recover the entire €124,500 reagent outlay of the period. A short sensitivity analysis frames the same point from the cost side. At unit reagent prices of €200, €250 and €300, total expenditure over the 498 runs would be €99,600, €124,500 and €149,400, of which €19,400, €24,250 and €29,100 (invariably 19.5%) would fall on negative runs, and the cost per actionable result would be €248, €310 and €373, respectively. Diagnostic-stewardship gating acts on this denominator: removing only half of the negative runs would save approximately €12,000 over twenty-two months at the current price and lower the cost per actionable result to about €281, without discarding a single informative test. The economic case for the panel is thus robust to plausible variation in reagent price but highly sensitive to the proportion of non-actionable runs—precisely the variable that gating is designed to control. These per diem and episode figures are illustrative local assumptions rather than measured costs (Section 4.6). The direction of the trade-off can be made explicit as a break-even calculation. At an assumed general-ward cost of €600–800 per inpatient day, the €310 reagent cost per actionable result is recovered by averting on the order of 0.4–0.5 inpatient days per case; at intensive-care daily costs the break-even threshold falls below 0.25 days. Because coupling rapid panels with antimicrobial stewardship has been shown to shorten time to optimal therapy by 17–34 h and, in several series, to reduce length of stay by one or more days, the strategy is plausibly cost-saving even under conservative assumptions. This remains a modelled inference pending locally measured length-of-stay data: it is the avoidance of even a fraction of an excess inpatient day, rather than the reagent price itself, that determines whether the strategy pays for itself.

### 4.2. Pathogen and Resistance Epidemiology

The predominance of the genus-level *Staphylococcus* spp. target, co-reporting with *S. epidermidis* and carrying *mecA/C* in the great majority of cases, is the expected signature of coagulase-negative staphylococci in blood cultures and immediately raises the question of clinical significance versus contamination (Section 4.3). The Gram-negative profile, led by *E. coli* and the *K. pneumoniae* group, mirrors the organisms that drive both community- and hospital-onset bacteraemia. The resistance findings carry the greatest clinical weight: *CTX-M* and *KPC* together accounted for the majority of Gram-negative resistance detections and were most often co-reported with (though, in polymicrobial specimens, not genetically assignable to; Section 4.3) *E. coli* and *K. pneumoniae*. Early genotypic flagging of these determinants is where rapid panels are most useful, because it can prompt earlier use of an active agent days before phenotypic susceptibility is available [8,11]. In practical terms each determinant maps onto a specific therapeutic consequence: *mecA/C* and the MRSA signature to a glycopeptide or another anti-MRSA agent, *CTX-M* to a carbapenem (or a carbapenem-sparing option under stewardship), and *KPC*, *OXA-48-like* and the metallo-β-lactamases to the newer β-lactam/β-lactamase-inhibitor combinations or to cefiderocol. This means that even an inferential gene-to-drug linkage can shorten the interval to an active regimen (Table 3).

### 4.3. Interpretive Limitations of a Qualitative Assay

The most important caveat for the reporting clinician is that BCID2 is a qualitative assay. It returns “detected/not detected” for each target and provides neither an organism load nor a cycle-threshold value, which has several practical consequences. It cannot, on its own, distinguish true bacteraemia from contamination or from carry-over of nucleic acid from non-viable organisms; for coagulase-negative staphylococci, the single most frequent detection in our series, this distinction is exactly the one clinicians most need, and it must still be made on clinical and quantitative-culture grounds. The absence of a quantitative read also prevents any judgement of relative abundance in polymicrobial specimens: when *E. coli*, the *K. pneumoniae* group and *Proteus* spp. are co-reported, the panel cannot indicate which organism is the dominant pathogen and which is a minor or transient component. A qualitative result also cannot be used to monitor the trajectory of an infection. We therefore consider the routine provision of a semiquantitative metric (for instance, a binned cycle-threshold or a relative-signal indicator) to be a priority, because it would allow the contribution of each detected pathogen in a co-infection to be weighted rather than merely enumerated.

A second, related limitation concerns resistance genes. By regulatory design the panel reports a resistance gene only when a plausible carrier organism is also detected, and it does not establish the genetic linkage between the two [2]. In a monomicrobial specimen this is rarely problematic. In a polymicrobial specimen it is a genuine interpretive hazard: a report of *mecA/C* in a bottle growing both *S. aureus* and *S. epidermidis* does not reveal which species is methicillin-resistant, and a β-lactamase gene reported alongside two co-detected *Enterobacterales* cannot be assigned to one of them with certainty. Such configurations are common in our data (Section 3.3). Clinicians must therefore avoid two errors: assuming that a detected gene is expressed phenotypically by every co-detected organism, and assuming that the absence of a targeted gene equals susceptibility. The panel interrogates only a closed set of determinants and is blind to resistance mechanisms outside that set, as well as to organisms outside the 43 targets, which is the likely explanation for a large share of our 19.5% negative runs.

In day-to-day practice these reports are not released in isolation. At our centre a positive BCID2 result is telephoned to the treating team and simultaneously flagged to the clinical microbiologist and the antimicrobial-stewardship team, and the written report carries a standard interpretive comment stating that, in polymicrobial specimens, a detected resistance gene cannot be assigned to an individual co-detected organism and that phenotypic susceptibility testing will follow. Management then follows a simple local protocol: a staphylococcal *mecA/C* or MRSA signature prompts continuation or initiation of an anti-MRSA agent; *CTX-M* directs therapy toward a carbapenem (or a carbapenem-sparing agent under stewardship); *KPC* and *OXA-48-like* detections trigger ceftazidime–avibactam, while the metallo-β-lactamases *NDM* and *VIM* call for a ceftazidime–avibactam-plus-aztreonam combination or cefiderocol; *vanA/B* directs enterococcal therapy to linezolid or daptomycin; and a solitary coagulase-negative staphylococcal detection without a compatible clinical picture is handled as a probable contaminant pending repeat cultures. In every case empirical choices are revised once phenotypic susceptibility is available, which remains the arbiter for de-escalation. A formal audit of concordance between BCID2 and conventional subculture was outside the scope of this retrospective analysis; in routine use, on-panel identifications agreed with subculture in the great majority of specimens, and the discordance that did occur was largely confined to the 19.5% of off-panel/negative runs and to additional organisms—chiefly anaerobes and off-panel non-fermenters—recovered only by extended culture. The frequency of such post-BCID2 discrepancies is itself a worthwhile target for prospective monitoring (Section 4.6).

### 4.4. The Need to Validate New and Complementary Tests

The high unit cost and the interpretive limits together make a strong case for local validation work rather than passive adoption, and three lines of work are warranted. The first is validation of test-selection (gating) algorithms that match panel use to the clinical questions where it changes management, so that the cost of negative and non-actionable results is minimised. The second is validation of lower-cost or tiered orthogonal approaches, such as targeted single-plex confirmatory PCR, rapid phenotypic antimicrobial-susceptibility platforms, or selective use of the panel only after a Gram stain consistent with a high-yield scenario. The third is validation of semiquantitative or quantitative read-outs against quantitative culture and clinical outcome, so that the contribution of each organism in a polymicrobial bacteraemia can be assessed. Each new or modified pathway should be validated locally against the standard-of-care reference before it is allowed to influence therapy, both because diagnostic accuracy is setting-dependent and because the economic argument that justifies a €250 test must be re-examined whenever the testing strategy changes.

### 4.5. Toward Direct-from-Primary-Sample Testing: Why a Reliable Culture-Free Multiplex Does Not Yet Exist

BCID2 is fast only after a bottle has signalled positive, yet the dominant delay in BSI diagnosis is not identification but the biological enrichment that precedes it: continuous-monitoring systems typically need 12–48 h, and longer for fastidious or low-burden organisms, to flag a bottle. An assay run directly on the primary blood specimen could in principle identify the pathogen within 3–6 h of venepuncture and would transform sepsis management [13,14]. Despite two decades of effort, however, no culture-free assay has matched the reliability of culture-dependent panels, and blood culture remains the reference standard; the obstacles are fundamental rather than incidental.

Two obstacles are decisive. The first is sensitivity: adult BSI is paucibacterial, often 1–10 colony-forming units (CFU)/mL and frequently below 1 CFU/mL, orders of magnitude below the limit of detection of conventional multiplex PCR (~10^3–10^4 copies per reaction) [13,15]. Blood culture overcomes this by biological amplification, whereas a culture-free assay must detect an unamplified target in the few microlitres of extract drawn from a sample inoculated, in the bottle, at 8–10 mL, and against a vast excess of human DNA and potent PCR inhibitors (haem, immunoglobulin, anticoagulants) that further depress the microbial signal unless host material is selectively depleted [14,16,17]. The second is specificity: assays that amplify free nucleic acid may report DNA from dead, cleared or translocated organisms (“DNAemia”), and because most patients investigated for sepsis do not have a true BSI (blood-culture positivity ~9%), even a low false-positive rate yields a poor positive predictive value [17]. Rapid identification also provides no phenotypic minimum inhibitory concentration, so a viable isolate is still required for full susceptibility testing and typing.

The platforms developed to date illustrate the difficulty. Broad-range 16S/18S rDNA PCR on whole blood (e.g., SepsiTest) adds adjunctive value but limited stand-alone sensitivity [18]; broad-range PCR with electrospray-ionisation mass spectrometry (IRIDICA) achieved wide identification directly from blood [19,20] yet was withdrawn from the market [14]. T2 magnetic resonance (T2*Candida*, T2Bacteria), the only system cleared for direct whole-blood detection, reaches a limit of detection near 1 CFU/mL and roughly 90% sensitivity for on-panel targets but only ~43% for any bloodstream pathogen, given a panel of six bacteria and five *Candida* species, and reports no resistance information [21,22,23]; plasma microbial cell-free DNA sequencing (e.g., Karius) is hypothesis-free and broad but costly, slower than a point-of-care multiplex, and limited by specificity, since non-causative cell-free DNA is common [24]. A recent meta-analysis of direct-from-blood molecular assays found pooled sensitivities of only 0.42–0.78, insufficient to replace culture [16]. No platform has yet reconciled the three requirements a primary-sample assay must meet at once—high sensitivity at paucibacterial loads, specificity that separates active infection from background DNA, and a broad panel that also reports resistance—at acceptable cost and turnaround. Until one does, biological enrichment in blood culture, and thus culture-dependent panels such as BCID2, will remain the backbone of BSI diagnosis; the realistic near-term gains lie in shortening the pre-analytical and incubation interval and in coupling these panels with stewardship rather than in replacing culture, and, as argued in Section 4.4, any culture-free assay must first be validated against culture locally before it is trusted to direct therapy.

### 4.6. Strengths and Limitations

The strengths of this study are its real-world, consecutive design over an extended period and its explicit pairing of microbiological epidemiology with an economic appraisal. Its limitations are those inherent to a single-centre, descriptive analysis: we did not link results to individual clinical outcomes, length of stay or antibiotic-consumption data, so the downstream savings that offset reagent cost are discussed rather than measured; we did not perform a head-to-head accuracy comparison against the phenotypic standard of care for each specimen, nor did we quantify the frequency of discordance between the panel and conventional subculture (a target we flag for prospective audit); and the genus- and family-level targets, together with the qualitative read-out, constrain the granularity of the epidemiological picture. In addition, coagulase-negative staphylococcal detections were not clinically adjudicated as true bacteraemia versus contamination or catheter-associated infection, so the burden attributed to these organisms may overestimate clinically significant bloodstream infection; genotype–phenotype agreement, the prevalence of multidrug-resistant isolates, and turnaround time relative to conventional methods were not quantified in this dataset; and pathogen frequencies are expressed at the level of detections and specimens. These limitations point to the same conclusion: a prospective, culture-reconciled and outcome-linked validation is required.

## 5. Conclusions

In a high-resistance southern Italian setting, the BCID2 panel provided rapid and broad identification of bloodstream pathogens and their resistance determinants, with a positivity of 80.5% and a resistance landscape dominated by *mecA/C*, *CTX-M* and *KPC*. These benefits are real but conditional. The strategy carries a high and partly unavoidable cost (nearly one reagent euro in five was spent on results that returned no on-panel target), and its qualitative output leaves two clinically important questions unanswered: the relative contribution of each organism in a co-infection, and the carrier of each resistance gene in a polymicrobial specimen. We conclude that the value of syndromic blood-culture testing is maximised only when it is embedded in a diagnostic-stewardship framework, supported by semiquantitative reporting, and accompanied by the local validation of complementary and lower-cost tests.

## Figures and Tables

**Table 1:** BCID2 testing volume and outcome by calendar segment, P.O. “Vito Fazzi”, July 2024–April 2026.

Period	Runs (*n*)	Single-Pathogen	Polymicrobial	Total Positive (%)	Negative (%)
Jul–Dec 2024	137	88	19	107 (78.1)	30 (21.9)
2025	282	189	41	230 (81.6)	52 (18.4)
Jan–Apr 2026	79	53	11	64 (81.0)	15 (19.0)
**Total**	**498**	**330**	**71**	**401 (80.5)**	**97 (19.5)**

Percentages for positive/negative are computed over runs in the same period.

**Table 4:** Direct-cost analysis at €250 per BCID2 run.

Item	Runs (*n*)	Cost (€)	Share (%)
Total testing	498	124,500	100.0
Actionable (positive) runs	401	100,250	80.5
Negative runs (cost of negatives)	97	24,250	19.5
July–December 2024	137	34,250	27.5
2025	282	70,500	56.6
January–April 2026	79	19,750	15.9

Shares for the three calendar segments are computed over total testing; downstream length-of-stay and antibiotic-consumption effects are not included.

## Data Availability

The aggregated data supporting the reported results are available from the corresponding author upon reasonable request, subject to institutional data-protection requirements.
